# Mitoproteomics: Tackling Mitochondrial Dysfunction in Human Disease

**DOI:** 10.1155/2018/1435934

**Published:** 2018-11-08

**Authors:** María Gómez-Serrano, Emilio Camafeita, Marta Loureiro, Belén Peral

**Affiliations:** ^1^Laboratory of Cardiovascular Proteomics, Centro Nacional de Investigaciones Cardiovasculares (CNIC), 28029 Madrid, Spain; ^2^Centro de Investigación Biomédica en Red sobre Enfermedades Cardiovasculares (CIBERCV), Spain; ^3^Proteomics Unit, Centro Nacional de Investigaciones Cardiovasculares (CNIC), 28029 Madrid, Spain; ^4^Instituto de Investigaciones Biomédicas Alberto Sols (IIBM), Consejo Superior de Investigaciones Científicas & Universidad Autónoma de Madrid (CSIC-UAM), 28029 Madrid, Spain

## Abstract

Mitochondria are highly dynamic and regulated organelles that historically have been defined based on their crucial role in cell metabolism. However, they are implicated in a variety of other important functions, making mitochondrial dysfunction an important axis in several pathological contexts. Despite that conventional biochemical and molecular biology approaches have provided significant insight into mitochondrial functionality, innovative techniques that provide a global view of the mitochondrion are still necessary. Proteomics fulfils this need by enabling accurate, systems-wide quantitative analysis of protein abundance. More importantly, redox proteomics approaches offer unique opportunities to tackle oxidative stress, a phenomenon that is intimately linked to aging, cardiovascular disease, and cancer. In addition, cutting-edge proteomics approaches reveal how proteins exert their functions in complex interaction networks where even subtle alterations stemming from early pathological states can be monitored. Here, we describe the proteomics approaches that will help to deepen the role of mitochondria in health and disease by assessing not only changes to mitochondrial protein composition but also alterations to their redox state and how protein interaction networks regulate mitochondrial function and dynamics. This review is aimed at showing the reader how the application of proteomics approaches during the last 20 years has revealed crucial mitochondrial roles in the context of aging, neurodegenerative disorders, metabolic disease, and cancer.

## 1. Introduction

Mitochondria represent the metabolic dynamism of the cell, and they are present in the cytoplasm of all eukaryotic cells relying on aerobic metabolism [1]. These intracellular organelles are involved in a number of vital processes including the generation of adenosine triphosphate (ATP) by oxidative phosphorylation (OXPHOS), the determination of the redox state through the generation and detoxification of reactive nitrogen and oxygen species (RNOS), the regulation of calcium homeostasis, and cell death [2, 3]. Mitochondrial activity comprises various processes, including the activity of the mitochondrial electron transport chain (ETC) complexes, substrate oxidation through the tricarboxylic acid (TCA) cycle and ATP synthesis, *β*-oxidation of fatty acids, ketogenesis, and biosynthesis of pyrimidines, heme groups, and urea [4, 5]. Additionally, mitochondria sense oxygen and nutrient levels, providing the energy required for other cellular processes such as muscle contraction or heat production, and maintain ionic gradients and membrane potentials in excitable cells, allowing the secretion of hormones and neurotransmitters [6].

Due to the huge number of cellular processes in which this organelle is implicated, even in the closed context of cellular respiration, the term mitochondrial function can refer to a variety of features. In fact, the definition of mitochondrial capacity has been broadened during the last decade to include additional features such as mitochondrial dynamics, which includes fusion and fission events, turnover (biogenesis and mitophagy), and plasticity [7]. The consideration of these mitochondrial capabilities has advanced our understanding of how the cell may respond to metabolic disturbances such as oxidative stress, inflammation, and gluco- and lipotoxicity [8], among others. Moreover, mitochondrial dynamics is influenced by energy expenditure and nutrient supply, and changes in mitochondrial architecture have emerged as a new mechanism of adaptation to metabolic demand [9]. Therefore, the mitochondrion is both origin and target of several metabolic signals which orchestrate cellular function and homeostasis [4].

Mitochondrial function can be tackled through *in vivo*, *ex vivo*, and *in vitro* methods, which have been extensively reviewed [7, 10–13]. In general, measuring aspects of mitochondrial function *in vivo* is preferable to *in vitro* assessments, although this approach might be less specific and even become unattainable depending on the type of sample under study. Despite that these *in vivo*, *ex vivo*, and *in vitro* approaches provide invaluable data on specific mitochondrial functions (e.g., submaximal and maximal ADP-stimulated OXPHOS capacity, TCA cycle flux, rates of fatty acid uptake, and RNOS production), they have limitations. Thus, the invasiveness of the method, the indirect measurement of the parameter, the impossibility to discriminate between mitochondrial and cytosolic shared functions, the expensiveness of the hardware/methodology, and/or the lack of data on mitochondrial density usually hamper the application of these methodologies in the study of mitochondrial function [7]. In addition, while the aforementioned approaches address mitochondrial functions individually, the central role of mitochondria in cellular metabolism and maintenance demands the application of additional innovative techniques that have the capacity to provide a global view of mitochondria in a single experiment. Although in an indirect manner, proteomics offers this functional snapshot thanks to the robustness, velocity, sensitiveness, and precision that mass spectrometry (MS) has acquired during the last decades. MS is recognized as the method of choice for both protein identification and quantification, and it has been the driving force for proteomics research development [14, 15].

Here, we describe innovative approaches to the quantitation of the mitochondrial proteome, with an emphasis on the oxidative modifications involved. In addition, we include cutting-edge experiments aimed at unveiling the mitochondrial interactome, since to deepen the role of mitochondria in health and disease it is essential to monitor not only the alterations to mitochondrial protein composition, but also how these proteins organize in interaction networks that regulate mitochondrial function and dynamics. The scope of the present review is to show the reader how the application of proteomics methodologies during the last 20 years has discovered key mitochondrial roles in the context of aging, neurodegenerative disorders, metabolic disease, and cancer.

## 2. The Mitochondrial Proteome

As a reminiscence of its prokaryotic past, the mitochondrion hosts its own genome. In mammalian cells, the mitochondrial DNA (mtDNA) only encodes 13 proteins belonging to the mitochondrial respiratory chain, 2 ribosomal RNAs, and 22 transfer RNAs, determining its intramitochondrial translation code, which is markedly different from the extramitochondrial one and is tightly regulated to maintain mitochondrial function [16]. The remaining mitochondrial proteins, i.e., the components of the TCA cycle, *β*-oxidation and protein transport, the other respiratory chain subunits, and the apoptotic factors, are all nuclear-encoded, which makes the definition of the mitochondrial proteome extremely challenging.

Since most mitochondrial proteins have to be imported into the organelle, there are several mechanisms whereby they can be transported [17, 18]. The import of cytosolically synthesized mitochondrial proteins usually requires cytosolic chaperones, such as heat shock proteins 70 and 90. These chaperones guide pre-proteins to mitochondrial outer membrane receptors (primarily TOM70 and TOM20) of the TOM (translocase of the outer membrane) complex [19]. The successive import into the diverse suborganelle compartments (that is, outer membrane (OM), intermembrane space (IMS), inner membrane (IM), or mitochondrial matrix) depends on several targeting signals recognized by various mitochondrial protein import complexes (reviewed in [17]). Of note, the characterization of key components and pathways involved in protein targeting has been extensively described over the last three decades, including oxidative folding machinery in the IMS, which contributes to the redox-dependent control of proteostasis [20]. Moreover, protein sorting could be differentially regulated among different organisms and physiological or pathological conditions [21], currently constituting an area of intensive research [20, 22, 23].

### 2.1. Key Aspects of the Mitochondrial Proteome

To date, the wealth of data that have largely contributed to define the mitochondrial proteome comes from proteomics studies performed in a number of eukaryotic organisms. In the human context, the majority of the studies have been carried out in connection with disease research [24]. Hence, in the last 20 years, the study of the mitochondrial proteome has elucidated several properties about this extraordinary organelle, some of which are outlined below.

#### 2.1.1. Evolutionary Origin and Mitochondrial Proteome Composition

Evidence suggests that current mitochondrion derives from an *α*-proteobacterial endosymbiont which transferred its DNA to the host nucleus [25]. It is estimated that only 15–20% of the current human mitochondrial proteome derives from the original endosymbiont DNA [26], although mammalian mitochondrial proteins are more conserved (nearly 75%) compared to the rest of cellular components (around 48% of evolutionary conservation) [27, 28]. In fact, phylogenetic profiling has been very useful in elucidating the function of several mitochondrial proteins [28, 29].

At present, the number of mammalian mitochondrial proteins ranges from 1100 to 1900 depending on the database and resource used. In the last decade, despite of the broad dynamic range (up to six orders of magnitude) that mitochondrial proteins exhibit, which hinders the detection of less abundant proteins, MS analyses have significantly contributed to the description of the mitochondrial proteome [30, 31]. These results have underscored remarkable quantitative differences across different tissues.

#### 2.1.2. Tissue Heterogeneity

Although the mitochondrial function is essential for almost all cell types (with the exception of red blood cells, which lack mitochondria), there are tissue-specific differences. Large-scale studies have provided valuable information about tissue or cell specificity. Thus, heart mitochondria are not only over 5-fold more abundant than adipose tissue mitochondria but also qualitatively different. In their seminal proteomics comparison across tissues, Mootha et al. found that mitochondria from different tissues shared approximately 75% of their components [32], and these data have been repeatedly confirmed with the subsequent extensive analyses of the organelle. As later revealed by Pagliarini and colleagues, mitochondria from developmentally related organs tend to share more proteins [28]. Out of the ∼1100 mitochondrial proteins they initially described, nearly half of them appear to be core components virtually found in all tissues; however, some of these pathways showed striking patterns of tissue diversity. According to their results, the most representative mitochondrial proteome could be found in skeletal muscle followed by heart tissue, since both proteomes include the least proportion of less abundant mitochondrial proteins [28, 33]. Interestingly, respiratory complexes I (CI), II (CII), III (CIII), and V (CV) are found in high abundance in all the tissues surveyed in contrast to complex IV (CIV), which seems to have a reasonable number of subunits expressed in a tissue-dependent manner. In fact, protein expression strongly differs in complex IV and large ribosomal subunits of mitochondria between heart and adipose tissue, most probably responding to the differences in the energetic demand between these tissues [28, 33].

Apart from the differences in ETC complexes, some mitochondrial proteins and isoenzymes are preferentially, or even uniquely, expressed in certain cell types. For example, uncoupling protein-1 (UCP1) is a distinctive marker of mitochondria from brown adipocytes [34]. This protein supports the nonshivering thermogenesis by the uncoupling of mitochondrial respiration which characterizes brown adipose tissue.

Remarkably, the characterization of tissue-specific mitochondrial proteomes has enabled the description of the biosynthetic capacities of different organs and the construction of flux balance analysis models [35–37]. These innovative studies represent the use of the mitochondrial proteome as a framework for systems physiology analyses [38–40].

#### 2.1.3. Dual Localization of Mitochondrial Proteins

Since different compartments can exhibit similar functions, it is not surprising that proteins can be dually localized in the cell. Generally, dual localization refers to those protein products from a single gene locus that are present in multiple subcellular locations [41]. In the case of mitochondria, it is noteworthy that they actively interact with other subcellular compartments like the endoplasmic reticulum, the nucleus, or the peroxisomes, which favours the dual localization of proteins. Thus, it has been estimated that around 15–30% of mitochondrial proteins are dual-localized [30, 42]. This dual localization can be achieved through several mechanisms, including alternative splicing of the same gene product or alternative start sites from the same gene locus, which produce distinct proteins. Additionally, some proteins may intermittently interact in the surface of mitochondria (e.g., hexokinase binds to the channel protein VDAC1 in order to release intramitochondrial ATP for glucose phosphorylation [43]), while others may translocate from the mitochondria to the cytosol in certain conditions (a typical example is the release of cytochrome c under proapoptotic conditions [44]) or vice versa (e.g., BID protein transfers from the cytosol into the mitochondrion after cell death stimulus [45]). In addition, certain proteins can localize to two different compartments under similar physiological conditions (e.g., the enzyme complex pyruvate dehydrogenase, which participates in the TCA cycle in the mitochondria, whereas in the nucleus it produces histone acetylation as part of the epigenetic regulation [46]).

In this complex scenario, the reports on the mitochondrial localization of proteins not usually associated with mitochondria or the localization of well-known mitochondrial proteins outside the organelle must be carefully considered. The validation of these results could be quite challenging and should involve at least two independent methods. In this regard, quantitative proteomics analysis of the total proteome and the corresponding mitochondrial subproteome could reveal even subtle quantitative differences originated by dual localization of mitochondrial proteins [24]. Additionally, other resources such as The Human Protein Atlas (http://www.proteinatlas.org) could provide orthogonal evidence to proteomic results based on subcellular localization data obtained through antibody-based techniques [47].

#### 2.1.4. The Mitochondrial Subproteome

As expected from cellular complexity, not only organellar localization but also the precise compartmentalization within the proper organelle is important for protein function, adding an additional level of complexity to the study of the mitochondrial proteome. For instance, the IMS is key to ATP production by storing the electromotive force generated by the ETC, which in turn localizes to the OM (and proteins localized to membranes have very different properties from those found in the mitochondrial matrix or the cytosol). Nevertheless, up to date only a few proteomic studies have analysed the human mitochondrial subproteome. In this regard, Rhee and colleagues have greatly contributed to the description of the mitochondrial matrix [38] and IMS proteomes [48] by applying MS-compatible tagging techniques as APEX [49]. Among other advantages, APEX strategy selectively tags proteins that localize to any specific region in living cells, circumventing the requirement for organellar purification when applied to membrane-bound compartments. This *in vivo* system is based on ascorbate peroxidase (APX) activity, which produces biotin-phenol radicals that can conjugate to the side chain of amino acids in proteins only in its immediate proximity. Thus, once biotin-labelled proteins are obtained, they can be pulled down and subsequently analysed by MS [49]. Thanks to this approach, Rhee and coworkers described 31 [38] and 9 proteins [48] that had not been previously ascribed to the mitochondrion.

### 2.2. Mitochondrial Databases

Due to the critical roles that mitochondria play in both cell life and cell death, lots of efforts have been dedicated to obtaining a complete map of mitochondrial proteins from different tissues and organisms. To date, the application of MS methods has been the most successful strategy to define the mitochondrial proteome. Pioneering studies combining two-dimensional electrophoresis (2-DE) with MS methods date back to 1998 and 2001, with the analysis of mitochondrial extracts from human placenta [50] and a human neuroblastoma cell line [51], respectively. In 1999, MITOP database was released [52]. It was the first database for both nuclear- and mitochondrial-encoded proteins, comprising genetic and functional information on five species (including mouse and human), with annotated data derived from a variety of online resources and literature. Three years later, Taylor et al. published the first comprehensive human mitochondrial proteome, extending previous information about mitochondrial proteins identified by MS [53]. This study constituted the first step for the achievement of the MitoProteome Project (http://www.mitoproteome.org/) [54]. In its initial release, the Human Mitochondrial Protein Database contained 847 human mitochondrial protein sequences derived from public sequence databases and MS analyses [54]. This set was substantially revised in 2015, and, in the current version (as of February 2016), it covers information for 1705 genes and 3625 proteins. Additional reference databases have been released that list mitochondrial proteins identified via multiple approaches, including MS analysis, literature curations, and bioinformatics evaluations [55]. Some examples are MitoP2 [56] or MitoMiner [57]. The latter contains green fluorescence protein (GFP) localization images, is updated regularly, and provides useful lists of functionally related genes in up to 12 different organisms [58]. Currently, MitoMiner describes 1837 human proteins, providing experimental or bioinformatic prediction for mitochondrial localization. In 2008, Pagliarini et al. reported the largest compendium of mammalian mitochondrial proteins to date [28], the MitoCarta database (http://www.broadinstitute.org/pubs/MitoCarta), which encompassed 1098 genes coding for mouse mitochondrial proteins unveiled by experimental MS-based identification, bioinformatics analyses, and literature curation. This inventory was updated in 2016 after the use of improved transcript models, MS search algorithms, new database versions, and homology detection methods [27]. The current version (MitoCarta 2.0) consists of 1158 human and 1158 mouse genes encoding mitochondrial proteins whose localization and distribution across 14 tissues are provided [27].

Other interesting sources for mitochondrial annotation currently available are the UniProt database (http://www.uniprot.org) [59] and the Gene Ontology (GO) functional repository (http://www.geneontology.org) [60]. Recently, large-scale studies like the Human Proteome Map (HPM) initiative (http://humanproteomemap.org/) [61], the Human Protein Atlas (http://www.proteinatlas.org) [47], and COPaKB (the Cardiac Organellar Protein Atlas Knowledgebase) (http://www.heartproteome.org/) [62] have also provided highly specific new data on the subcellular localization of mitochondrial proteins.

## 3. Addressing Mitochondrial Dysfunction through High-Throughput Proteomics Techniques

**Figure 1:**
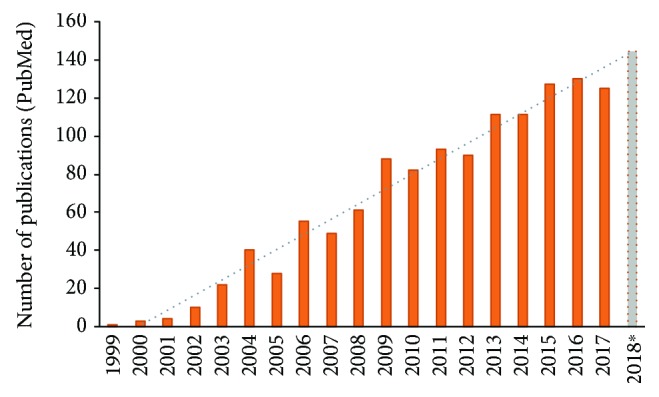
Timeline for “mitoproteomics” publications. Bars show the number of publications per year corresponding to the keywords “mitochondria” and “proteomics” in PubMed database (https://www.ncbi.nlm.nih.gov/pubmed). Note that the first proteomic study of human mitochondria [50] is not indexed. Results for 2018 are not included since they should be constantly updated during the writing process. According to the linear progression (dashed line), in 2018 we will probably reach a total of 144 publications related to mitochondrial proteomics (^∗^grey bar).

It is agreed that the first attempt to address the mitochondrial proteome was carried out by Rabilloud and colleagues 20 years ago [50]. Since then, the interest on mitochondrial proteomics has gradually increased, as reflected by a simple query on the PubMed database (https://www.ncbi.nlm.nih.gov/pubmed) (Figure 1). The publication of landmark proteomics studies describing the mitochondria of human [53, 63], rat [64, 65], mouse [28, 66], and yeast [67, 68] species, as well as those related to different tissues [32] and cells [69], have critically contributed to the definition of the mitochondrial proteome to date.

### 3.1. Isolation of Mitochondria

The starting point in the study of the mitochondrial proteome is mitochondrion isolation. Mitochondrion-enriched fractions have been used as starting material until differential centrifugation [70] became the method of choice. Three key steps can be distinguished in mitochondrial enrichment: (i) permeabilization and disruption of plasma membrane and release of mitochondria without compromising their integrity, (ii) differential centrifugation to remove cellular debris and larger organelles, and (iii) high-speed centrifugation to pellet the mitochondria [71]. The first step is usually performed through mechanical methods (e.g., mortar and pestle) under well-controlled buffer compositions. Some of the disadvantages of mechanical methods are excessive heat generation and stress on mitochondria, as well as increased intersample variability when executed manually [71]. In contrast, differential centrifugation should not compromise the quality or the yield of the preparation. Furthermore, additional gradient centrifugation protocols can be applied after differential centrifugation to obtain mitochondria with higher purity [72].

There are currently a few well-established protocols for isolating mitochondria [71, 73–75], although certain sample types might require specific procedures, as is the case of brain [76], adrenal glands [77], and adipose tissue [78]. Of note, the alternative use of magnetic devices for mitochondrial enrichment has gained popularity in recent years [78, 79].

The most common limitation of isolation strategies is the presence of carried-over nonmitochondrial proteins in the mitochondrial extracts, a phenomenon that is expected by virtue of the physical connection between the mitochondrion and other subcellular organelles [80]. However, high coverage of the total cellular proteome can be achieved thanks to recent improvements in protein identification methods based on liquid chromatography coupled with mass spectrometry (LC-MS) [24]. When combined with bioinformatics sorting of mitochondrial proteins, these data on protein identification reduce the necessity of purified mitochondria, which points out the combination of high-throughput MS analyses and database knowledge as a successful strategy, as previously described [78].

### 3.2. Sample Preparation for Mitochondrial Proteomics

As the identification of mitochondrial proteins is technically challenging due to their number and dynamic range, sample fractionation is desirable to reduce the complexity before MS analysis. While the first mitochondrial proteomics studies resorted to protein separation by 2-DE and peptide mass fingerprinting MS analysis to identify only a few dozen proteins [50, 51], later developments in LC-MS have facilitated the large-scale identification of mitochondrial proteins. In 2003, Taylor et al. identified 615 mitochondrial proteins from human heart by LC-MS (instead of 2-DE) analysis of 11 sucrose fractions [53], which represented a breakthrough in the identification of mitochondrial proteins. Later that year, gel filtration chromatography was used to separate mitochondrial proteins from brain, heart, kidney, and liver of C57BL6/J mice [32]. From each tissue, the authors obtained 15–20 fractions that were subjected to LC-MS analysis, which allowed the identification of 133 proteins not previously associated with this organelle. However, Mootha et al. did not overlook that their analysis was biased toward abundant molecules and that improvements in sample preparation, protein separation, and sensitiveness of MS were necessary. Many researchers have resorted to protein separation by sodium dodecyl sulfate (SDS) polyacrylamide gel electrophoresis (PAGE) followed by gel slicing and analysis of each band by high-performance LC-MS [28, 64, 66, 81]. Other less frequent fractionation methods have been applied prior to LC-MS, like the aforementioned sucrose gradients [53] and gel filtration chromatography [32].

### 3.3. Identification of Mitochondrial Proteins

Most proteomics experiments involve the enzymatic digestion of a complex protein extract prior to peptide separation, ionization, and analysis by LC-MS. For the last 15 years, LC-MS in data-dependent acquisition (DDA) mode has been the standard for proteomics identification [82, 83]. DDA is a milestone for high-throughput proteomics due to its efficiency at identifying peptides and proteins. In this method, peptide peaks (precursor ions) that overcome a threshold intensity in a survey MS scan are selected for fragmentation in the MS^2^ mode. This fragmentation generates an experimental fragment ion pattern characteristic of the peptide amino acid sequence that can be matched against theoretical patterns derived from protein databases for peptide identification [84]. In spite of the power of the DDA method, random peptide sampling for fragmentation entails a bias towards those with the strongest signal, which hampers the reproducible quantitation of especially low-abundance peptides.

Conversely, targeted acquisition methods, most notably selected reaction monitoring (SRM) [85], focus the mass spectrometer capabilities on the fragmentation of a limited number of peptides representing proteins of interest with very high sensitivity and quantitative accuracy. However, owing to the limited fraction of the proteome amenable to SRM analysis, this method is not suitable for most proteomics experiments, where detailed protein composition is required. So far, only a few studies have resorted to targeted methods for the study of human mitochondrial protein profiles [86, 87]. Other authors have applied targeted methods to the study of such posttranslational modifications (PTMs) as acetylation [88, 89] and phosphorylation [90, 91] in the mitochondrial proteome.

In recent years, data-independent acquisition (DIA), which combines the advantages of DDA and SRM, has raised increasing interest. The DIA method performs the fragmentation of all the peptides within a defined mass-to-charge (*m*/*z*) window; the window is then progressively shifted until the full *m*/*z* range is covered. This enables accurate peptide quantitation without being limited to profiling a predefined set of peptides of interest, but at the cost of losing the link between precursor and fragment ions thereof. An implementation of the DIA method, SWATH, relies on spectral libraries that collect *m*/*z* values, relative intensities, retention times, etc., to trace the so-obtained compound fragmentation maps for specific peptides. Since these libraries must be carefully built up based on additional DDA experiments [92], only a few studies have relied on SWATH to address mitochondrial disturbances in disease models [93–95]. A recent development based on DIA, DiS (data-independent scanning), resorts to narrower isolation windows to facilitate peptide identification based on the conventional database search methods developed for DDA [96]. DiS has been successfully used to generate a highly detailed structural map of OXPHOS mitochondrial supercomplexes in several models, including the characterization of protein species and PTMs thereof that regulate complex and supercomplex assembly [96]. DIA methods have enormous potential for mitoproteomics, where highly curated databases (e.g., MitoCarta 2.0) will help improve mapping and quantification of mitochondrial proteins.

### 3.4. Quantification of Mitochondrial Proteins

Depending on the separation strategy selected, protein quantification can be performed through gel-based or MS-based (*gel-free*) approaches. In gel-based approaches, the quantification of differentially abundant proteins is conducted by comparison of gel images, and MS methods are set aside for protein identification. Paramount among gel-based strategies is difference gel electrophoresis (DIGE), an improvement on 2-DE in which the proteins from two samples are labelled with different fluorescent dyes and separated in the same gel, allowing a more accurate relative quantification as compared to 2-DE [97].

In contrast, *gel-free* strategies rely on mass spectrometric data for both protein identification and protein quantitation. At this point, it should be noted that *gel-free* approaches have higher yield and well-established advantages in quantification over gel-based techniques. The quantification method in MS-based approaches includes label-free and stable isotope labelling (SIL) methods. Most often, label-free quantification relies on the counting of MS^2^ spectra from the peptides of a given protein [98]. The label-free approach has been used in numerous large-scale studies, as it provides a simple way to carry out relative comparisons across different samples with improved dynamic range of quantification [99]. Conversely, in SIL strategies, the relative quantification is based on the addition of chemically equivalent differential mass tags, which allow the comparison of up to ten samples in a single experiment. Despite that some limitations have been pointed out for SIL-based quantification, namely, limited linear dynamic range, complex sample preparation, and high cost of the reagents [100], this approach has great advantages in quantification accuracy and reproducibility. Some examples of SIL techniques successfully employed in comparative mitoproteomics are (i) metabolic labelling with stable isotope labelling by amino acids in cell culture (SILAC) [101, 102], (ii) enzymatic labelling with ^18^O during proteolytic digestion [103, 104], and (iii) chemical labelling employing isotope-coded affinity tags (ICAT) [105, 106], isobaric tags for relative and absolute quantitation (iTRAQ) [78, 107–109], or tandem mass tags (TMT) [110, 111].

### 3.5. Mitoproteomics in Human Disease

Early mitoproteomics studies relied mainly on gel-based techniques for protein separation, such as 2-DE, in combination with a variety of MS methods. After 2-DE separation, the protein spots of interest must be sliced off from the gel and digested for later off-line MS analysis. In these pioneering studies, the underrepresentation of extreme pI, low abundant, or very hydrophobic proteins, which has been pointed out as an important drawback inherent to 2-DE, has hindered the identification of a higher number of mitochondrial proteins [50, 65, 112].

During the first decade after mitoproteomics implementation, alternative separation methods based on SDS-PAGE coupled with LC-MS were developed that allowed significant advances in the investigation of the mitochondrial proteome (reviewed in [100]). In the last decade, there has been a dramatic increase in the number of publications related to mitoproteomics (e.g., until 2008, the total of publications according to PubMed indexing was 273, a number that was overcome in just the last two years) (Figure 1).

Most mitoproteomics studies have been performed on animal models, as these offer more possibilities for mitochondrial isolation and purification. Results on these models give insight into basic functions of proteins and reveal the mechanisms of adaptation under loss of function of the respective proteins (e.g., knockout models), altered nutritional status (e.g., obesity), pharmacological treatment, or physiopathological stressors, shedding light on mitochondrial responses. In the case of human specimens, there are significant limitations regarding sample availability, some of which are related to obvious difficulties in collecting the material, whereas others derive from medical and ethical issues. Despite this bottleneck, during the last years the increased robustness of MS approaches and the application of multiplexing methods (such as iTRAQ or TMT), which substantially reduce the amount of sample needed, have counteracted the limitations inherent to working with human samples, bringing mitoproteomics closer to translational research [78].

Furthermore, the combination of mitoproteomics with other high-resolution techniques has provided new insights into mitochondrial disease. For instance, Walheim and coworkers have recently described the combination of quantitative mitochondrial respirometry (through Seahorse technology) and proteomics (LC-MS-based total protein approach) to understand how protein changes relate to mitochondrial energy capacity during diet-induced obesity [113]. This approach, termed “respiromics,” has shown not only that CI concentration controls mitochondrial lipid oxidation in the liver, but also that obesity affects the functional capacity of this complex by regulating the nuclear- but not mitochondrial-encoded subunit concentrations.

In the following sections, the reader will find a selection of recent illustrative mitoproteomics studies in different pathological contexts, emphasizing the global perspective obtained on mitochondrial research thanks to these cutting-edge techniques.

#### 3.5.1. Aging

Mitochondrial dysfunction is considered one of the hallmarks of aging [114], where several aspects of mitochondrial physiology are involved, such as mitochondrial biogenesis and turnover, calcium dynamics, energy sensing, RNOS production, and apoptosis [115]. In addition, aging is recognized as an independent risk factor for different pathologies such as cardiovascular disease (CVD), type 2 diabetes mellitus (T2DM), neurodegenerative disorders, or cancer [116, 117]. Several mitoproteomics studies have addressed the effect of aging in the context of these pathologies: see [78, 118] for metabolic disease, [119–121] for cardiac disturbances, and [122, 123] for neurodegenerative disorders. Downregulation of complex subunits and mitochondrial respiratory capacity is a common feature of normal aging mitochondria, although particular changes have been highlighted depending on the model, the age differences, and the particular tissue under study. For instance, in C57BL/6 mice, CI subunits have been described both to decrease [124] and to increase [123] with aging, and Stauch et al. have reported different patterns of metabolic and ETC modifications according to the age of mice in both synapses [125] and whole-brain mitochondria [126]. In the context of obesity, a pioneering study on human adipocyte mitochondria showed a general decrease in metabolic enzymes and ETC subunits in older compared to younger individuals, despite that all individuals were middle-aged (from 32 to 52 years) [78].

Of note, mitochondrial dysfunction has been addressed by proteomics in mouse models of premature aging (also known as Hutchinson-Gilford progeria syndrome (HGPS)) [127]. Although the genetic components of this disease are well-known (i.e., a dominant mutation in the LMNA gene leading to the expression of progerin), the mechanisms underlying cellular damage, senescence, and accelerated aging in HGPS are only partly understood. Resorting to SILAC and LC-MS analysis, the authors observed a pronounced downregulation of several components of the mitochondrial ATPase complex together with an upregulation of glycolytic enzymes. Interestingly, the analysis of different tissues revealed that mitochondrial dysfunction was time- and dose-dependent in the HPGS mouse models [127]. Altogether, these results underline the tight regulation undergone by mitochondria with aging, pointing out the importance of the surrounding context for this organelle.

#### 3.5.2. Neurodegenerative Diseases

As previously introduced, mitoproteomics studies in the context of the nervous system have been closely related to aging research. This fact most probably responds to the observation of a decline in brain energy metabolism with aging [128]. This metabolic change is considered part of the physiological aging contributing to age-related neurodegeneration (e.g., Alzheimer's disease (AD) or Parkinson's disease (PD)), where different evidences support a key role of mitochondria in its progression [123, 129, 130]. Several proteomics approaches, including redox proteomics (see *Redox Mitoproteomics* Section in this review), have pointed out specific mitochondrial proteins as key regulators, providing new insights into their role in aberrant mitochondrial function and structure in AD and PD, among others (for an excellent review, see [131]). Interestingly, alterations to TCA enzymes and OXPHOS subunits are an early feature of mitochondrial dysfunction that can be observed prior to the deposition of significant amyloid plaques and neurofibrillary tangles in AD [132]. Causal links for mitochondrial abnormalities and oxidative damage in synaptic degeneration and amyloid-*β* deposition have been proposed [133, 134]. Moreover, substantial differences between synaptic and nonsynaptic mitochondria have been previously reported [135, 136]. In the mitoproteomics field, Graham et al. have recently evidenced distinct molecular fingerprints between mitochondrial subpopulations that are likely to influence the synaptic morphology *in vivo*, highlighting mitochondrial CI as an upstream regulator in neurodegenerative pathologies [137]. Thus, mitoproteomics research is regarded as a promising tool for the investigation of preclinical and clinical biomarkers of neurodegeneration and its potential treatments [131].

#### 3.5.3. CVD

Heart tissue is rich in mitochondria and highly dependent on the catabolism of branched-chain amino acids and fatty acids within this organelle. Mitochondrial function has been closely related to cardiomyopathies [138] and ischemic heart disease [139]. Although the precise underlying mechanisms are not fully understood, it is clear that mitochondrial metabolism is heavily involved in both cardiac injury [140] and cardiac protection [141].

Heart ischemia causes tissue injury through an acute phase followed by secondary damage. Notably, ischemic preconditioning (brief episodes of ischemia preceding prolonged ischemia) provides robust protection against cell death resulting from ischemia and reperfusion (I/R) injury in the heart and is currently explored as a new target for cardiac treatment [142]. In fact, a combination of mitoproteomics with respiratory assays and electron microscopy (EM) described a “mitochondrial preconditioning” independent of cytosol that confers protection against I/R-induced respiratory failure and oxidative damage [143].

The cardiac mitochondrial proteome has been widely explored in models of I/R [144–147]. Most of these studies have also relied on redox approaches (see *Redox Mitoproteomics* Section in this review), since mitochondrial RNOS generation has been described as a double-edged sword contributing to myocardial infarction and stroke as well as ischemic preconditioning [148].

#### 3.5.4. Metabolic Disease: Obesity and Diabetes

In essence, obesity consists in an excessive fat accumulation due to an imbalance between energy expenditure and uptake. Sedentary lifestyle and overnutrition have greatly contributed to the present epidemic of obesity, which is also aggravated by the aging of population [149]. Mitochondrial dysfunction has long been associated to the development of insulin resistance (IR) and T2DM, since changes in physical activity and overnutrition have a direct impact upon mitochondrial function [4]. During the last decades, most mitoproteomics studies addressing IR and T2DM pathologies were based on skeletal muscle samples, therefore overlooking the relevance of mitochondria in adipose tissue and the systemic implications of its impairment (for an excellent review of mitoproteomics in the context of metabolic disease, see [118]). Most of these studies relied on animal or cellular models, which imposed additional limitations to the extent of their conclusions [150]. The key role for white adipose tissue in regulating energy expenditure and IR was clearly recognized during the first decade of the 2000s [5, 151], but by that time the only comparative study between white and brown adipocyte mitochondria had been carried out in mice [102]. Therefore, the study of human adipocyte mitochondria was of outmost importance.

**Figure 2:**
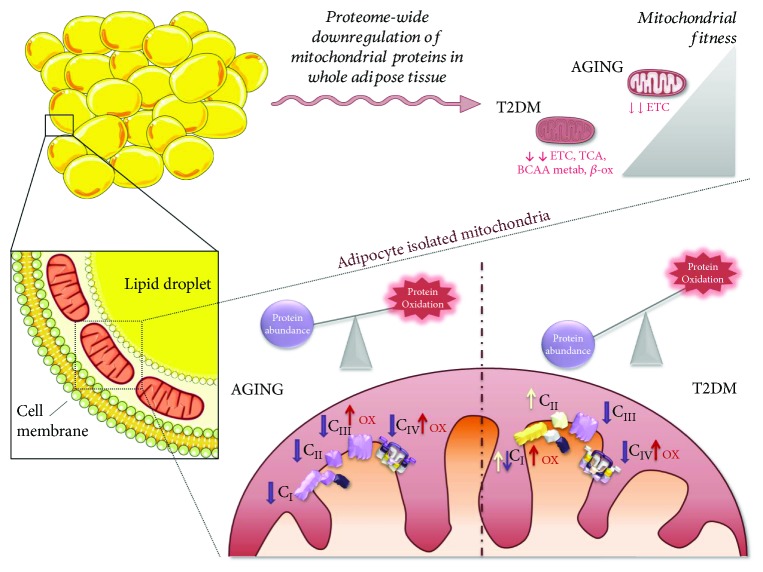
High-throughput proteomics for the study of adipose tissue mitochondria. Gomez-Serrano et al. carried out a deep study of mitochondrial alterations in human fat. In a first study based on whole adipose tissue samples, they described proteome-wide downregulation of the identified mitochondrial proteins in both aging and T2DM processes thanks to the application of systems biology analyses (*upper panel*). Alterations in relation to aging were mostly circumscribed to the electron transport chain (ETC), whereas disturbances in diabetic patients included also other metabolic pathways such as tricarboxylic acid (TCA) cycle, branched-chain amino acid (BCAA), metabolism, or *β*-oxidation, most likely resembling a decrease in mitochondrial mass with severe consequences on mitochondrial fitness [155]. In a second study, the authors proceeded to the isolation of adipocyte mitochondria in order to further analyse this organelle (*lower panel*). Resorting to a redox approach, they described thiol oxidative modifications as well as protein abundance changes [78]. The authors found that thiol protein oxidation was inversely correlated to protein levels in adipocyte mitochondria and that this relationship was more dramatic in T2DM compared to the aging process. Additionally, OXPHOS mitochondrial- *vs*. nuclear-encoded protein modules were altered in T2DM (note that complex modules are represented in different colours to indicate upregulation (yellow) and downregulation (purple), respectively). Thus, their results underscored defects in respiratory capacity and protein import in aging and T2DM. Of note, CIV emerged as a common target of oxidative damage connecting aging and T2DM development. Graphical elements from this figure were taken and adapted from Servier Medical Art Powerpoint image bank (http://smart.servier.com/). Servier Medical Art by Servier is licensed under a Creative Commons Attribution 3.0 Unported License.

In 2015, Lindinger et al. published the first mitoproteomics study on this area [152]. Despite the limitations of their analysis (e.g., only 62 putative mitochondrial proteins were identified), the authors validated four proteins (citrate synthase, mitofilin, HADHA, and LETM1) that were inversely associated with body mass index (BMI) in white adipocyte mitochondria from 76 patients. Their results confirmed previous studies regarding whole adipose tissue biopsies [153, 154]. A few years later, Peral and colleagues published two complementary works that constitute the most comprehensive depiction of the human adipocyte mitochondrial proteome to date. In their first study, they described a global reduction of mitochondrial proteins (~150 proteins) in whole visceral adipose tissue biopsies from T2DM individuals by means of a high-throughput multiplexed proteomics approach based on iTRAQ technology [155]. In their second study, the authors deepened the proteome changes in adipocyte mitochondria with an improved redox approach [78] (see *Redox Mitoproteomics* Section for further details). Their results revealed impaired assembly of mitochondrial complexes together with defective protein import as a new potential cause of mitochondrial dysfunction in T2DM (Figure 2). In fact, the authors underlined the alteration of mitochondrial import pathways at different levels, like the TOM complex or the mitochondrial intermembrane space assembly (MIA) pathway [78], extending their previous results regarding the alteration of heat shock protein 70 in diabetic patients [154]. Additionally, the multiplexing capacity of their proteomics approach allowed the authors to address also the differences associated to aging in the same experiment, pointing out CIV as a common target for mitochondrial remodelling in both the aging and T2DM processes [78].

#### 3.5.5. Liver Disease

Also, in close relation to aging or metabolic pathologies, we could find excellent works performed in the context of liver disease. Nonalcoholic fatty liver disease (NAFLD) is a chronic liver condition strongly associated with obesity, IR, and T2DM [156]. It can gradually progress to nonalcoholic steatohepatitis (NASH) in 12–40% of cases, cirrhosis (15% of NASH patients), and hepatic cancer (10%) [157], and oxidative damage on mitochondria has been tightly related to this fatal progression [158]. Thus, NAFLD and NASH attract a great interest among the scientific proteomics community (reviewed elsewhere [159]). These studies have revealed the importance of mitochondrial proteins in liver disease together with enzymes involved in methionine metabolism [160, 161], whose alteration may lead to an increase in both protein and lipid oxidative damage influencing the development of NAFLD and NASH. Furthermore, the beneficial action of metformin treatment in NAFLD has been also revealed in a mitoproteomic study of a mouse model [162].

Interestingly, ethanol-induced stress on liver has been also addressed through these approaches [163, 164], revealing an important modification of the mitochondrial proteome under these xenobiotic stimuli.

#### 3.5.6. Cancer

Since the mitochondrion is the powerhouse of the cell, this organelle has long been related to carcinogenesis, cancer progression, and metastasis, although its specific role is still disputed [165]. It has been suggested that mitochondrial pathways such as RNOS signalling or Ca^2+^ homeostasis could play a critical role in the alteration of energy metabolism in cancer cells [166, 167]. Besides, the Warburg effect [168], a shift from mitochondrial respiration towards glycolysis in cancer cell metabolism, constitutes a canonical observation for almost all cancer types whose mechanism remains obscure. Of note, the wealth of data provided by cancer mitoproteomics has provided new insights into the Warburg effect (outstandingly reviewed in [169]), although proteomic studies in cancer have been mostly used for tumor subtyping [170].

The heterogeneity of tumor tissue and cell composition hampers the analysis of the corresponding mitochondria, hindering the interpretation of the results. Notwithstanding the above, altered structure and protein levels have been reported for mitochondria from cancer cells, and mitochondria have also been found affected by antioncogenic treatments (reviewed in [170]). Interestingly, a recent study of Casal's group has revealed the mechanisms leading to OXPHOS downregulation in colorectal cancer [171]. By using SILAC technology, subcellular fractionation, LC-MS analysis, and bioinformatics, the authors described spatial proteome alterations at the subcellular level that help elucidate the molecular mechanisms underlying colorectal cancer metastasis [171]. Apart from mitochondrial dysfunction, their data suggest that proteins belonging to the TCA cycle and OXPHOS might have secondary localizations such as the nucleus and the cytoskeleton, confirming previous results in MCF7 breast cancer cells [172].

#### 3.5.7. Other Pathologies

Because of the great interest in mitochondrial proteomics shown in the last decade, many examples of models or diseases addressed through these approaches can be found. Thus, proteomics studies have also revealed the importance of mitochondrial proteins in hypertension [173], osteoarthritis [174], and male infertility [175, 176], among others.

## 4. A Further Step: Redox Mitoproteomics

Since mitochondria contain major producers of RNOS, including components of the respiratory chain and other redox enzymes [177], together with powerful antioxidative defence systems [178], measuring redox damage in this organelle is central to deciphering disease pathogenesis. Thus, significantly increased mitochondrial RNOS levels have been associated with CVD [179–181], cancer [182, 183], neurodegenerative disease [184, 185], and aging [55].

Among many other chemically feasible oxidative PTMs that may occur in proteins, redox reactions involving Cys thiol (-S-H) groups are frequent, mostly reversible, enzymatically controlled, and selective *in vivo* [186, 187] and are considered the main way whereby oxidants integrate into cellular signal transduction pathways [188]. Common reversible Cys oxoforms include disulphide bonds (-S-S-), S-glutathionylation (-S-SG-), S-nitrosation (-S-NO), and S-sulfenylation (-S-OH), whereas sulfinic (S-O_2_H) and sulfonic acid (S-O_3_H) constitute irreversible Cys oxoforms (except for eukaryotic 2-Cys peroxiredoxins, where sulfinic acid may be reduced by sulfiredoxin [189]). Despite that the mechanisms that regulate these processes in the mitochondrion remain obscure, the respiratory chain should play an essential role, as it harbours the main producers of mitochondrial RNOS, CI [190], CII [191], and CIII [192]. Still, the development of redox mitoproteomics, i.e., the large-scale characterization of the specific mitochondrial Cys residues that sense the oxidative milieu, has been obstructed by the lability and low abundance of Cys oxoforms, which for decades could only be tackled by site-directed mutagenesis [193]. Proteomics approaches to protein Cys thiol oxidation provide a variety of methods for the enrichment, detection, and quantitation of such modifications, which can be classified into gel-based and gel-free methods.

### 4.1. Gel-Based Redox Mitoproteomics

Reversible S-nitrosation (-S-NO) affects a number of mitochondrial proteins and is thought to be a major way in which nitric oxide (NO) metabolism modulates mitochondrial physiology [194]. Galkin et al. used fluorescein-N-ethylmaleimide (NEM) labelling and Blue Native (BN) separation to identify by MS the specific residue of mitochondrially encoded ND3 subunit that becomes accessible to chemical modification only in the deactive form of mitochondrial CI from bovine heart mitochondria [195]. This enzyme had been previously suggested to undergo modification by S-nitrosation under pathological conditions during hypoxia or when the NO : O_2_ ratio increases [196]. Under these conditions, RNOS and NO generation by the respiratory chain complexes and NO synthases, respectively, could cause the accumulation of the endogenous nitrosating agent peroxynitrite and result in permanent deactivation of the enzyme [196]. Shortly afterwards, S-nitrosation of the Cys switch of CI active/deactive transition was associated with marked cardioprotective effects in mice [197]. In 2007, Hurd et al. adapted DIGE [97] for the detection of proteins differentially oxidized by mitochondrial RNOS in mitochondria isolated from rat heart [198]. Based on the oxidation of a small subset of mitochondrial thiol proteins in the absence of bulk thiol changes, the authors suggested a possible link between mitochondrial RNOS production and the modulation of mitochondrial fatty acid and carbohydrate metabolism. Chouchani et al. further developed the DIGE method for the selective analysis of S-nitrosated proteins in mitochondria from rat heart and liver, where S-nitrosated proteins could be discriminated from proteins oxidized due to NO metabolism [199]. The authors identified 13 S-nitrosated mitochondrial proteins plus four that were oxidized, probably due to evanescent S-nitrosation relaxing to a reversible thiol modification. Interestingly, S-nitrosation was found to selectively and reversibly inhibit the activity of enzymes central to mitochondrial metabolism, namely, aconitase, mitochondrial aldehyde dehydrogenase, and *α*-ketoglutarate dehydrogenase. The contribution of NO-mediated S-nitrosation to the nerve injury-evoked pathology was shown in mouse spinal cord by Scheving et al., who used DIGE to reveal abundance changes of S-nitrosated proteins after sciatic nerve injury upon pretreatment with a NO synthase inhibitor [200]. A DIGE-based study also enabled quantitation of S-nitrosation in mitochondrial CI in normoxic and ischemic intact mouse hearts [201], where S-nitrosation had been associated with marked cardioprotective effects [197].

It has been suggested that changes to the sulfenome, which encompasses Cys sulfenic (-S-OH) groups derived from the initial oxidation of Cys thiol (-S-H) groups, play a central role in redox signalling. Thus, identifying the sulfenome under oxidative stress has been pointed out as a way to detect potential redox sensors. To date, the sulfenome has only been addressed in *Arabidopsis thaliana* cell suspensions exposed to H_2_O_2_ stress [202–204]. Using the dimedone derivative 4-(pent-4-yn-1-yl)cyclohexane-1,3-dione as a probe, Akter et al. identified by MS more than 200 sulfenylated proteins after the oxidative treatment of *Arabidopsis thaliana* cells, 14 of which located to the mitochondrion and were involved in the TCA cycle and the ETC [203].

### 4.2. MS-Based Redox Mitoproteomics

Early MS-based redox proteomics approaches resorted to the so-called biotin switch assay, whereby labile S-nitrosated Cys are converted to stable biotinylated Cys and the corresponding proteins purified by affinity chromatography and detected by immunoblotting or MS [205–208]. This assay allowed Hao et al. to pinpoint by LC-MS 68 S-nitrosation sites from 56 rat cerebellar proteins after treatment with S-nitrosoglutathione [205]. Some of these S-nitrosated Cys were involved in important mitochondrial processes like the formation of the permeability transition pore, the inactivation of sarcomeric creatine kinase, and mitochondrial relocation and neuroprotective activity of protein/nucleic acid deglycase DJ-1 [205]. The biotin switch assay has also been adapted for the analysis of additional Cys oxoforms (e.g., sulfenic acid [209–211]). Moreover, MS-based identification of S-glutathionylated proteins isolated from mouse liver helped to reveal a potential mechanism of resistance to acetaminophen-induced hepatotoxicity [212]. A combination of chemical enrichment methodologies with MS allowed Gould et al. to map 2596 sites of Cys modification including S-nitrosylation, S-glutathionylation, S-acylation, and S-sulfenylation, in wild-type mouse livers under normal physiological conditions [213]. Structural analysis localized these modifications in unique, evolutionary conserved protein segments outside commonly annotated functional regions.

**Figure 3:**
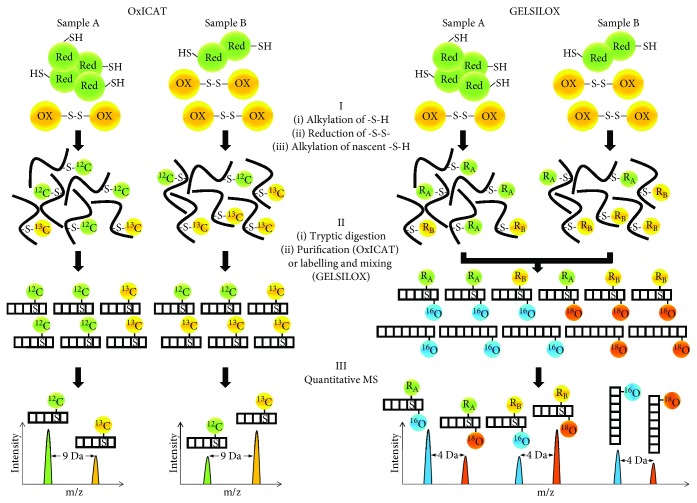
The OxICAT and GELSILOX approaches to redox proteomics. *Step I*: a hypothetical protein, which exists in two different samples A and B in both the reduced (green) and oxidized (disulfide-linked, yellow) form, is incubated with an alkylating agent (^12^C-IAM for OxICAT, *left*, and, e.g., IAM, for GELSILOX, *right*) to block free sulfhydryl groups. Then, a reducing agent is used to cleave the disulfide bonds and the nascent thiol groups blocked using a second alkylating agent (^13^C-IAM for OxICAT and, e.g., NEM, for GELSILOX). *Step II*: the proteins are digested with trypsin and the so-obtained peptides either affinity-purified (OxICAT) or tagged with ^18^O labelling prior to mixing (GELSILOX). *Step III*: two independent quantitative MS assays reveal the extent of thiol modification in samples A and B (OxICAT), whereas one single MS assay yields not only the relative amount of the reduced and oxidized forms but also the relative quantitation of the corresponding protein between the two samples based on the MS signals from noncysteine peptides.

Quantitative LC-MS approaches to Cys oxidation often start by stabilizing the redox status of the biological sample, usually by blocking free thiols with highly thiol-reactive reagents (e.g., iodoacetamide (IAM)). Thereafter, reversibly oxidized Cys residues are treated with a strong reducing agent like dithiothreitol (DTT) and the so-obtained free thiols alkylated with a second reagent to enable specific detection of Cys oxoforms. Differential Cys tagging with SIL reagents produces either chemically indiscernible light and heavy or isobaric peptides, which coelute in the chromatographic separation and exhibit identical ionization properties. Quantitation is then achieved by measuring the intensity of either neighbouring heavy and light precursor mass signals in the MS spectrum or reporter ions in the MS^2^ spectrum [214–218]. Weerapana et al. used click chemistry conjugation and affinity enrichment to characterize Cys functionality in mouse and human proteomes based on the levels of Cys oxoforms, some of which pertained to mitochondrial proteins [219]. In 2008, Leichert et al. proposed the OxICAT method [220], based on the 2-plex ICAT (isotope-coded affinity tag) technology, to address the thiol redoxome (Figure 3, *left*). A comparative study of redoxome analysis based on DIGE [198] and OxICAT identified 63 proteins in mouse heart that were prone to thiol oxidation upon H_2_O_2_ treatment, many of which were mitochondrial TCA cycle members [221]. Results showed superior sensitivity of the OxICAT approach, which has been progressively used for investigating the thiol redoxome in a variety of nonmitochondrial biological samples, including *Saccharomyces cerevisiae* [222], *Schizosaccharomyces pombe* [223, 224], *Escherichia coli* [225], *Caenorhabditis elegans* [226], *Phaeodactylum tricornutum* [227], *Drosophila melanogaster* [228], and *Mus musculus* heart [180]. Of note, Go et al. investigated the effect of acute cadmium exposure on the redox proteome and metabolome of mitochondria isolated from mouse liver [229]. OxICAT unveiled significantly increased oxidation relative to controls in 1247 Cys residues from 547 proteins that were mapped to mitochondrial pathways like OXPHOS and pyruvate metabolism. A recent work by Topf et al. used OxICAT to quantify oxidation in more than 4300 Cys residues pertaining to ~2200 proteins from *S. cerevisiae* upon exogenous and intracellular mitochondria-derived oxidative stress [230]. Site-specifically mapping these redox-active Cys to the cellular translation machinery helped the authors identify redox switches for global translation modulation by mitochondrially produced RNOS. SIL labelling of peptides derived from S-glutathionylated mouse liver proteins based on 6-plex TMT has evidenced that this Cys modification has a fundamental role in energy metabolism [231]. Chouchani et al. also resorted to TMT-based, 6-plex peptide tagging to label reversibly oxidized thiols in mouse brown adipose tissue, where mitochondrial RNOS induction was identified as a mechanism that supports UCP1-dependent thermogenesis and whole-body energy expenditure [232]. Recently, alterations to S-glutathionylated mitochondrial protein levels were linked to fatiguing contractions in mice [233]. For that, the authors resorted to selective reduction with a glutaredoxin enzyme cocktail and resin-assisted enrichment followed by 10-plex TMT labelling of total thiol and S-glutathionylated peptides to unveil about 2200 S-glutathionylation sites.

These quantitative, MS-based methods have enabled not only the identification of redox-regulated thiol proteins, most of them of mitochondrial origin, but also to pinpoint the precise reactive Cys and to quantify their oxidation level. Nevertheless, their dependence on trapping and enrichment of Cys-containing peptides prevents parallel assessment of alterations in protein abundance. Given that redox signalling or oxidative stress could trigger changes in protein abundance owing to protein degradation or protein translocation, their assessment is essential to providing an integrated view of the underlying processes.

### 4.3. The GELSILOX Approach

To circumvent the above-described deficiencies, in 2012, Martínez-Acedo et al. proposed GELSILOX (gel-based stable isotope labelling of oxidized Cys, Figure 3, *right*) [234]. By combining differential alkylation of reduced and oxidized Cys with SIL of both Cys-containing and non-Cys peptides, GELSILOX provides simultaneous quantification of reversible Cys oxoforms and protein abundance. For that, the method relies on a statistical framework newly developed for SIL-based quantitative proteomics, the weighted spectrum, peptide, and protein (WSPP) model [235]. GELSILOX was initially applied to characterize the main redox targets of H_2_O_2_ in human endothelial cells as well as to study alterations to the thiol redoxome in mitochondria isolated from rat heart cardiomyocytes after the I/R insult [234]. The method was later applied by Ruiz-Meana et al. to show that decreased subsarcolemmal mitochondrial respiration in heart rat after I/R is paralleled by increased Cys oxidation, with ischemic preconditioning preventing both phenomena [143]. In their investigation of the role of altered mitochondrial function in aging, Fernandez-Sanz et al. resorted to GELSILOX to demonstrate increased oxidation of different subunits of mitochondrial ATP synthase in hearts from old mice upon I/R [120]. Moreover, GELSILOX allowed Guaras et al. to hypothesize that specific Cys oxidation is linked to the differential stability and degradation of mitochondrial CI during reoxygenation. For that, the authors assessed the oxidation state of more than 1000 Cys residues from 784 mitochondrial proteins of mouse fibroblasts [96]. Of note, a combination of redox approaches allowed Gomez-Serrano et al. to point out defects in protein translocation to the mitochondrion as responsible for mitochondrial dysfunction in T2DM and aging. For that, the authors relied on a GELSILOX-based method to quantify 244 Cys oxidation sites within a set of 116 *bona fide* mitochondrial proteins that evidenced for the first time a link between increased thiol oxidation and decreased protein abundance under both conditions (Figure 2).

Despite tremendous advances of recent years, several challenges remain to be addressed by redox mitoproteomics. First, some of the aforementioned methodologies have been demonstrated appropriate for *in vitro* studies, and therefore their suitability to tackle the mitochondrial redoxosome *in vivo* is in question. For this reason, and also because redox proteomics studies based on human mitochondria are still scarce, the experimental evidence for physiological readouts of mitochondrial Cys thiol oxidation is yet limited. In this regard, care must be taken if reagents with widespread use like DTT and dimedone are incorporated to *in vivo* assays, as the former can stimulate RNOS production by mitochondrial enzymes [236] and the latter can react rapidly with sulfenyl amides [237]. Another hurdle to overcome when investigating the mitochondrial redoxome is the isolation of the organelle, which has traditionally been carried out by ultracentrifugation [238] and has the drawback of carrying over organelles with similar size profiles like lysosomes. In this regard, the effective enrichment of mitochondria is often a lengthy procedure, which is detrimental to the preservation of oxidative PTMs. Furthermore, as stated above, prevalent methods like OxICAT fail to provide parallel assessment of protein abundance changes in the same assay that unveils the alterations of Cys oxoforms, which hampers data interpretation.

Finally, notwithstanding the importance of oxidative modifications, many other PTMs take place in the mitochondrion that are responsive to the stressful and changing environmental conditions in which this organelle exerts its functions, e.g., phosphorylation, acetylation, ubiquitination, and succinylation. The functional significance of these PTMs and their interplay in the mitochondrion are still under investigation.

## 5. The Mitochondrial Interactome

Another step forward on defining the mitochondrial proteome was to understand the roles and consequences of protein-protein interactions (PPIs), as proteins do not usually function in isolation but interact with other molecules. Understanding PPIs is fundamental for the development of systems biology and novel therapeutics. A number of public databases provide comprehensive lists of physical PPIs in human as well as in other species [239]. The STRING (Search Tool for the Retrieval of Interacting Genes/Proteins) database (http://string-db.org/) contains both known and predicted protein-protein interactions [240]. IntAct (http://www.ebi.ac.uk/intact) and MINT databases provide molecular interaction data and curation tools for annotation [241]. Both IntAct and MINT are active contributors to the IMEx consortium (http://www.imexconsortium.org), which provides a nonredundant set of physical molecular interaction data from a broad taxonomic range of organisms. The Database of Interacting Proteins (DIP, http://dip.doe-mbi.ucla.edu/dip/) is an integration of experimentally determined PPIs on different organisms and contains an interesting visualization tool which provides a graphical illustration of these interactions [242]. In The Human Protein Reference Database (HPRD) the information of PPI is manually curated from the published literature and it also integrates the information of domain architecture, PTMs, interaction networks, and disease association for each protein in the human proteome [243].

The information about the physical interactions contained in these and other nonmentioned databases was derived from several methodologies applied to dissect PPIs. Methods based on immunoprecipitation or two-hybrid system in combination with Western blotting have been widely used to identify interaction partners of specific proteins. However, these approaches are limited to a reduced number of proteins.

The recognition that proteomes are highly interconnected networks, or interactomes, has changed biology's view of cause and effect, simultaneously highlighting the importance of systems biology and necessitating its use in deciphering complex biological phenomena for the development of novel therapeutics [64, 244–246]. This concept of connectivity modulating systems explains how functionally dissimilar systems, such as tissues, can have largely similar compositions [61, 247, 248].

In this regard, the combination of affinity purification and MS (AP-MS) has emerged as a powerful approach to delineate biological processes. The method consists in tagging a bait protein with an affinity tag such as His-tag, flag-tag, or tandem affinity purification (TAP) tag for expression *in vivo*. The group of partners interacting with the bait protein can be purified from the cell lysate by affinity or immunoaffinity techniques and analysed by MS for identification of the interacting components [249]. This technique has been applied to study interactions in a high-throughput manner [246, 250, 251], but also subsets of specific networks have been analysed, such as the interaction partners of COX4 for the study of CIV assembly [252], the characterization of ATPase protein components [253], and the interaction partners of the inner mitochondrial membrane protein LETM1 [254]. In the neurodegeneration research field this technique has unveiled parkin-associated proteins that are implicated in the mechanism of mitophagy of defective mitochondria [255], and a map of mitochondrial proteins involved in neurodegenerative diseases was obtained in 2017 [256]. Other recent works have described the bcl-2 interactome in human lung adenocarcinoma cells [257], as well as the interaction network of a set of 50 mitochondrial proteins that lacked significant functional annotation [258] to gain insight into their role in health and disease.

However, the largest set of AP-MS experiments to date is Bio-Plex [259, 260], consisting of more than 5891 individual experiments with the human ORFeome (the collection of open reading frames (ORFs)). The data can be explored through the web application Bio-Plex (http://bioplex.hms.harvard.edu/index.php) [261]. This platform allows the exploration of individual networks, highly interconnected network communities, subthreshold interactions, and specific experimental information like peptide spectral match counts for individual AP-MS experiments [260].

In spite of the power of AP-MS for studying interactions, it still suffers from some limitations. The global study of PPIs in a given system requires tagging every ORF of interest to provide a measurable readout or to enable purification and identification of the protein complex. In addition, protein tagging can be time-consuming and can disrupt interactions or alter the localization of the protein complex [262]. Moreover, these approaches generally trigger perturbations to the system, such as genetic modification of the native cells or disruption of the original cellular context, which increases the rate of both false-positive and false-negative results [263]. The selection of few cell types under artificial expression conditions also poses limitations to the interactome characterization [246, 259]. For these reasons, current networks remain of limited use in interpreting biological phenomena, as the observed interactions may not reflect the interactome landscapes within other systems or in disease. Alternative approaches are necessary for mapping native, endogenous interactomes under multiple conditions [264], as well as to survey the global organization of protein complexes within mitochondria. For the last years, protein complexes have gathered attention, as proteins arrange to form macromolecular assemblies that facilitate their function and regulation. Although macromolecular complexes are essential players in numerous biological processes, a limited number of methods suitable for defining the composition and functional dynamics of such assemblies have been described. Thus, the comprehensive mapping of these complex networks of stable and transient associations remains a key goal.

To study macromolecular complexes, it is necessary to apply mild, nondestructive techniques during sample processing to obtain the native complexes. Density gradient centrifugation, size exclusion chromatography (SEC), and native electrophoresis have been applied to define large multiprotein complexes, e.g., the respiratory chain supercomplexes in mitochondria [265, 266] and pyruvate dehydrogenase and other complexes from chloroplasts [267]. Density gradient centrifugation requires large protein amounts and suffers from low resolution. SEC provides a robust workflow for the separation of cytoplasmic complexes; however, it is not compatible with membrane complexes as they are extremely sensitive to the separation conditions [264, 268, 269], and size exclusion columns lead to sample dilution and cannot be used for complexes larger than 5 MDa [270]. Notwithstanding these drawbacks, interesting results have been achieved (e.g., the combination of quantitative proteomics and SEC to map 291 coeluting complexes, where the use of triplex labelling enabled monitoring of interactome rearrangements [262]). Due to these reasons, BN electrophoresis has been the preferred method for fractionating mitochondrial membrane protein complexes. Below is a description of the methods available for the study of the mitochondrial interactome.

### 5.1. BN-PAGE

BN-PAGE was developed by Schägger and von Jagow [271] and was firstly used for the isolation and characterization of the respiratory complexes from bovine mitochondria [272, 273]. BN electrophoresis is a special case of native electrophoresis for high-resolution separation of enzymatically active protein complexes from tissue homogenates and cell fractions, between 10 and 10,000 kDa [274]. Higher resolution can be attained by decreasing the pore size in the polyacrylamide gradient gel. The principle of BN-PAGE is simple, yet powerful: the protein complexes interact with Coomassie dye through hydrophobic interactions and, since the dye is negatively charged, become negatively charged as well. With this negative charge, the protein complexes migrate, at neutral pH, towards the anode, the same way that SDS forces proteins to run towards the anode in SDS-PAGE [249]. The difference between SDS-based SDS-PAGE and Coomassie-based BN-PAGE is that SDS is a strong denaturing agent, while Coomassie dye does not affect the integrity of the native protein complexes. Therefore, due to the external charge induced by Coomassie dye, BN-PAGE separates native protein complexes according to their molecular mass in the 100–1500 (or even higher) kDa mass range. BN-PAGE provides a vast amount of information: complex size, subunit composition and stoichiometry, assembly of protein complexes into supercomplexes, and relative abundance and stability of subcomplexes within protein complexes. In addition, the relative abundance of these protein complexes can also be determined [249].

BN electrophoresis in combination with MS analysis [275] emerged as one of the methods of choice for the analysis of the “complexome” (protein complex proteome) when in 2009 Wessels and coworkers reported the first “complexome profiling” [265]. The authors analysed a mitochondrial enriched fraction from a human HEK cell line and identified 48 of the 71 canonical subunits of the respiratory chain complexes (CI–CIV). Manually cut slices from BN gels were subjected to label-free, semiquantitative LC-MS, and protein abundance profiles were determined across the gel. Protein correlation profiling (PCP) was performed to identify potentially interacting proteins. Later that year, another work confirmed the potential of this technique studying how the composition and formation of respiratory chain complexes change under anaerobic conditions in mitochondrial protein extracts from *S. cerevisiae* [276].

### 5.2. PCP-MS-Based Approaches

PCP is based on the generation of individual protein elution or migration profiles, where comparison with other profiles by computational clustering and other approaches allows the identification of putative interacting proteins on the basis of similarities in their elution profiles [277, 278]. Since its discovery, lots of works performed on mitochondria have been published [264, 270, 279–282], and the methodology has been gradually improved: first, regarding the quality of native gel separation and gel slicing [281], and second, based on the evolution of mass spectrometers, which are faster and more sensitive [283].

Combined PCP-MS approaches have been used to characterize mitochondrial complexomes, leading to the discovery of TMEM126B as an assembly factor of respiratory chain CI [270], the analysis of human mitochondrial ribosomal complexes [282], the identification of novel assemblies of voltage-dependent anion channels/porins and TOM proteins [281], the analysis of changes occurring in cytoplasmic and mitochondrial interactomes in response to apoptosis initiation by caspase activity [264], the description of the assembly mechanism for CI [280], and the superassembly of CIII and CIV [279]. Although the aforementioned works performed the correlation profiles based on protein migration in a BN gel, elution profiles derived from other techniques, such as biochemical fractionation or SEC, are also feasible [262, 284, 285].

A potential advantage of the PCP approach is that hundreds to thousands of protein complexes can be simultaneously and rapidly analysed. A disadvantage of the PCP approach is that currently only soluble complexes with interactions that are not markedly weakened by the buffers used can be analysed [278].

### 5.3. Alternative Techniques: An Insight into Cross-Linking

Finally, another outstanding MS approach that can provide new evidences on the systems-wide organization of proteins in intact mitochondria is cross-linking mass spectrometry (XL-MS). In XL-MS, native protein contacts are captured using a cross-linker, which is typically a small organic molecule composed of a spacer arm and two functional groups that are reactive toward specific residue side chains. After proteolytic digestion of the cross-linked sample, residue-to-residue cross-links can be localized by MS-based peptide sequencing. A cross-link can only occur if the cross-linker can bridge the distance between the residues. Detected cross-links, therefore, reveal maximum residue-to-residue distance constraints within and in between proteins, providing insights into protein conformations, protein complex architectures, and protein interaction networks [286].

Although the identification of cross-linked peptides is not trivial even for purified protein complexes, which may be available in large quantity [263], recent advances in cross-linking methods have enabled high-throughput identification of protein interactions in complex mixtures and living cells [287], allowing, e.g., the determination of the molecular structure of the mammalian mitochondrial ribosome [288]. XL-MS provides data about the location of the interaction site in the biomolecule under study, an information that cannot be derived from the other methods for studying PPIs. Many works have contributed to improving (i) methods to enrich cross-links [289–291], (ii) cross-linking chemistries [263, 292–297], (iii) detection and identification strategies [292, 296, 298–305], and (iv) tools to perform structural analysis based on cross-linking sites [306–310]. The most widely used cross-linkers are active esters, such as disuccinimidyl suberate and bis(sulfosuccinimidyl) suberate, which induce nucleophilic attacks on primary amines and thus rely on the coupling of Lys residues [311].

Recent works have performed high-throughput investigation of protein structures and interactions at the mitochondrial proteome-wide level using XL-MS approaches [286, 287]. By combining cross-link identifications from 11 mitochondrial preparations from 4 different tissues, Schweppe et al. reported 1920 unique residue-to-residue connections and found intercomplex cross-linked peptides, supporting the existence of the respirosome in an excellent agreement with cryo-EM models [287]. Very recently, Liu et al. analysed intact mouse heart mitochondria and identified 3322 unique residue-to-residue contacts, the largest survey of mitochondrial protein interactions reported so far. In addition, the authors revealed the mitochondrial localization of four proteins not yet included in the MitoCarta database [286].

The major limitation of XL-MS from the structural point of view is that it cannot directly determine the relative stoichiometry of subunits in a complex, although this information can be derived from complementary quantitative MS methods. In addition, the field remains heterogeneous, with various experimental protocols and software applications, and this variety may seem intimidating to newcomers. Nevertheless, XL-MS studies of partially purified complexes will certainly provide new insights about protein interaction networks and their changes on perturbation, e.g., as a result of mutations connected to diseases. If this technique becomes optimized for complex samples, it will solidify the relevance of cross-linking-based methods not only in structural biology but also in systems biology, thus advancing the convergence of structural and cell biology [311].

## 6. Outlook

These are exciting times for exploring the human mitochondrial proteome. Since this highly active organelle contains major producers of RNOS, encompassing redox enzymes and OXPHOS proteins, measuring the redox damage with innovative redox proteomics-based approaches will be crucial to puzzle out human pathology in the near future. Mitochondria and energy metabolism are key components in life and death regulation. It is not surprising that dysfunctional mitochondria lie beneath the physiological process of aging and the unhealthy aging. Despite that research efforts have resulted in significant advances in the knowledge of the mitochondrial proteome, the identification of low-abundance proteins and the detection of very subtle expression changes under human physiological and pathological conditions is still challenging. In the coming years, improved MS instrumentation together with the combination of advanced labelling and fractionation methods will likely render a deeper view of the mitochondrial proteome that will shed light on the human diseases connected to mitochondria. So far, it is noteworthy that the application of systems biology analyses to big data sets accomplished through proteomics has greatly contributed to the depiction of the mitochondrial network. This achievement is particularly interesting, as it enables to assess whether and how protein abundance changes occur in a coordinated manner, a biological event that is well-known [312]. In the near future, interactomics will provide an upper level of information about complex regulation both inside and outside the mitochondrion, revealing the role of intricate protein networks in the whole cell metabolism. Thus, proteomics implementation for the study of the mitochondrial interactome constitutes a step forward that must be taken in the near future, especially in the context of human disease, where such kind of studies is scarce.

Furthermore, to understand the regulation of these proteins and networks, the high-throughput analysis of PTMs in health and disease is also of outmost importance. The unprecedented capacity of current MS-based approaches provides a vast amount of biologically relevant information, producing a molecular picture of the complex interplay between proteins and regulators. Of note, many PTMs have been described as molecular switches directly involved in protein activation and/or inhibition. Oxidative modifications constitute an exciting area of research due to the implications of RNOS in cell signalling and oxidative damage. Given that redox mitoproteomics has just started to be explored, the application of these approaches in different pathological models will probably elucidate novel mechanisms of redox regulation in human disease. Other PTM analyses have been performed in the mitochondrion, notably phosphorylation, acetylation, succinylation, and ubiquitination (reviewed in [313]); nevertheless, high-throughput studies have been hindered due to technical issues (e.g., usually only one, at most two, type of modification can be addressed in a single experiment). Therefore, the development of advanced algorithms for PTM discovery and their application in mitoproteomics will probably constitute a new milestone in mitochondrial research.

## Abbreviations

2-DE: Two-dimensional gel electrophoresis
AD: Alzheimer's disease
AP-MS: Affinity purification and tandem mass spectrometry
ATP: Adenosine triphosphate
BMI: Body mass index
BN: Blue native
CI: Complex I, NADH dehydrogenase
CII: Complex II, succinate dehydrogenase
CIII: Complex III, cytochrome c-oxidoreductase
CIV: Complex IV, cytochrome c oxidase
CV: Complex V or ATP synthase
CVD: Cardiovascular disease
DIA: Data-independent acquisition
DIGE: Difference gel electrophoresis
DiS: Data-independent scanning
DDA: Data-dependent acquisition
DTT: Dithiothreitol
EM: Electron microscopy
ETC: Electron transport chain
GELSILOX: Gel-based stable isotope labelling of oxidized Cys
GFP: Green fluorescence protein
GO: Gene ontology
HPLC: High-performance liquid chromatography
I/R: Ischemia/reperfusion
IAA: Iodoacetic acid
IAM: Iodoacetamide
ICAT: Isotope-coded affinity tag
IM: Inner membrane
IMS: Intermembrane space
IR: Insulin resistance
iTRAQ: Isobaric tags for relative and absolute quantitation
LC: Liquid chromatography
LC-MS: Liquid chromatography coupled with mass spectrometry
MIA: Mitochondrial intermembrane space assembly
MS^2^: Fragmentation spectrum
MS: Mass spectrometry
mtDNA: Mitochondrial DNA
*m*/*z*: Mass-to-charge
NEM: N-Ethylmaleimide
NO: Nitric oxide
OM: Outer membrane
ORF: Open reading frame
OXPHOS: Oxidative phosphorylation
PAGE: Polyacrylamide gel electrophoresis
PD: Parkinson disease
PCP: Protein correlation profiling
PPIs: Protein-protein interactions
PTM: Posttranslational modification
RNOS: Reactive nitrogen and oxygen species
SDS: Sodium dodecyl sulfate
SEC: Size-exclusion chromatography
SIL: Stable isotope labelling
SILAC: Stable isotope labelling by amino acids in cell culture
SRM: Selected reaction monitoring
T2DM: Type 2 diabetes mellitus
TAP: Tandem affinity purification
TCA: Tricarboxylic acid
TCEP: Tris(2-carboxyethyl)phosphine
TMT: Tandem mass tags
TOM: Translocase of the outer membrane
UCP1: Uncoupling protein-1
XL-MS: Cross-linking mass spectrometry.
